# Predictive value of left ventricular dyssynchrony for short-term outcomes in three-vessel disease patients undergoing coronary artery bypass grafting with preserved or mildly reduced left ventricular ejection fraction

**DOI:** 10.3389/fcvm.2022.1036780

**Published:** 2022-11-17

**Authors:** Jia Liu, Rui Fan, Cui-ling Li, Yan-qiu Liu, Dong-hong Liu, Wei Li, Feng-juan Yao

**Affiliations:** Department of Medical Ultrasonics, The First Affiliated Hospital of Sun Yat-sen University, Guangzhou, China

**Keywords:** left ventricular dyssynchrony, coronary artery bypass grafting, three-vessel disease, real-time three-dimensional echocardiography (RT-3DE), short-term outcomes

## Abstract

**Background and objective:**

Coronary artery bypass grafting (CABG) is the reference standard intervention in coronary artery disease (CAD) patients with three-vessel disease (3VD). We aimed to evaluate the predictive value of left ventricular (LV) dyssynchrony for short-term adverse outcomes in patients with 3VD undergoing CABG with preserved or mildly reduced LV ejection fraction (LVEF).

**Materials and methods:**

This study involved ninety-five 3VD patients with preserved or mildly reduced LVEF undergoing scheduled on-pump CABG. The pre-operative diameters and volumes of LV and LVEF were obtained by two-dimensional echocardiography. LV dyssynchrony parameters were acquired by real-time three-dimensional echocardiography (RT-3DE) and analyzed by HeartModel quantification software. And the perfusion index of LV was obtained by contrast echocardiography. The clinical endpoints of short-term adverse outcomes comprised 30-day mortality and/or composite outcomes of postoperative complications. Univariate and multivariate logistic regression analyses were used to identify risk factors for the occurrence of post-CABG short-term adverse outcomes.

**Results:**

Short-term adverse outcomes occurred in 12 (12.6%) patients. These patients had higher LV dyssynchrony parameters obtained through RT-3DE. The standard deviation (SD) of the time to minimum systolic volume (Tmsv) corrected by heart rate over 16 segments (Tmsv16-SD%) [odds ratio (OR), 1.362; 95% confidence interval (CI) (1.090–1.702); *P* = 0.006], one of the LV dyssynchrony parameters, was independently associated with short-term adverse outcomes. Patients with poor synchronization tended to spend more time in the intensive care unit (ICU) and hospital after surgery.

**Conclusion:**

Pre-operative LV dyssynchrony parameter Tmsv16-SD% obtained through RT-3DE could be a useful additional predictor of postoperative short-term adverse outcomes in 3VD patients with preserved or mildly reduced LVEF undergoing CABG.

## Introduction

Coronary atherosclerotic disease (CAD) is the most common heart disease and it remains a leading cause of death worldwide (1). CAD patients with three-vessel disease (3VD) have almost a twofold higher risk of mortality than patients with single-vessel disease (2, 3). As a safe and established intervention, coronary artery bypass grafting (CABG) is the standard care for 3VD patients (4, 5). Nevertheless, patients undergoing CABG remain at a high risk of adverse cardiovascular outcomes. In particularly, the highest risk exists during and shortly after surgery (6). Shroyer et al. (7) reported relatively high 30-day mortality and composite morbidity rates for CABG procedures (3.05 and 13.40%, respectively). Therefore, it is essential to identify risk factors to avoid short-term adverse postoperative outcomes after CABG.

The European System for Cardiac Operative Risk Evaluation (EuroSCORE) and its updated version EuroSCORE II are widely accepted risk models for cardiac surgery (8–10). In both models, left ventricular (LV) dysfunction is a validated risk factor, and its classification relies on the level of decrease in LV ejection fraction (LVEF). However, Jakobsen et al. (11) opined that depending on the measurement of LVEF obtained from echocardiography, in EuroSCORE, the model might have an unacceptable influence on risk classification. Some studies have identified that the EuroSCORE II model performs poorly in the low-risk patients and isolated cardiac surgery; it performs optimally in non-CABG surgery and in highest-risk patients (12, 13). In addition, most studies have focused on patients with severe LV dysfunction; however, patients with preserved or mildly reduced LVEF have usually been ignored. Therefore, additional indexes representing or associated with LV dysfunction could be potential and useful for post-CABG risk identification in 3VD patients with preserved or mildly reduced LVEF.

Left ventricular systolic dyssynchrony, which reflects the mechanical effects of asynchronous LV contraction, has been shown to be a prognostic indicator for some cardiovascular diseases and it also occurs in CAD patients (14–16). LV systolic dyssynchrony indexes can be acquired through real-time three-dimensional echocardiography (RT-3DE). This imaging technology can track myocardial trajectory in three-dimensional space and provide a quantitative and accurate evaluation of the synchronization (17).

Therefore, the main purpose of this study was to evaluate the predictive value of LV dyssynchrony parameters obtained from RT-3DE for short-term adverse outcomes after CABG in 3VD patients with preserved or mildly reduced LVEF. By documenting the advantages of this technology over conventional echocardiography, we hope that this study will find additional indexes besides LVEF and help to improve the standard of echocardiography care for 3VD patients before CABG in the future.

## Patients and methods

### Study population

Patients meeting the inclusion and exclusion criteria were enrolled from April 2018 to December 2021. The inclusion criteria were as follows: (1) CAD patients with 3VD confirmed by coronary angiography, waiting elective on-pump CABG; (2) LVEF >45% (Simpson’s method) (6, 18); and (3) sinus rhythm. The exclusion criteria were as follows: (1) the presence of chronic or acute pulmonary disease; (2) morbid obesity (body mass index >40 kg/m^2^); (3) moderate to severe valvular heart disease including stenosis, prolapse, and regurgitation; (4) cardiomyopathy; (5) previous pacemaker implantation; (6) prosthetic valve; (7) the presence of chronic or acute kidney disease; and (8) poor echocardiographic image quality. This study was registered at clinicaltrials.gov (NCT03235323) and was performed in accordance with the ethical standards of the Institutional Ethics Committee. Written informed consent was obtained from each patient. The enrolled patients underwent assessment consisting of demographic features, medical history, laboratory parameters, QRS duration, and echocardiographic imaging workup. Calculation of EuroSCORE II was done with the interactive calculator at www.euroscore.org.

### Two-dimensional echocardiography imaging

Echocardiography imaging was recorded in DICOM format using EPIQ7 Philips Ultrasound systems (Philips Medical Systems, Andover, MA, USA) and X5-1 transducer and 3.5-mHz probe were used. The standard examination was completed per the guidelines of the American Society of Echocardiography (19). LVEF was measured using modified biplane Simpson’s method from apical 4- and 2-chamber views. Parameters including LV end-diastolic diameter (LVDd), LV end-systolic diameter (LVDs), LV end-diastolic volume (LVEDV), LV end-systolic volume (LVESV), LV stroke volume (LVSV), and LVEF were recorded.

### Real-time three-dimensional echocardiography imaging and analysis

The RT-3DE full-volume datasets were acquired from the apical window of the entire LV. HeartModel acquisition (HMQ) mode, as a specific one-beat acquisition, was used to acquire 3DE datasets. At least three full-volume datasets throughout one cardiac cycle were obtained using this acquisition model. Gain and compression controls and time-gain compensation settings were optimized to ensure image quality. Data sets were acquired using a wide-angle acquisition (93 × 80 degrees) mode. Four wedge-shaped subvolumes (93 × 20 degrees each) were obtained from five consecutive cardiac cycles. Then, the digital echocardiographic videos were analyzed using fully automated quantification software (HeartModel, QLAB, version 2.0, Philips Medical Systems, Cleveland, OH, USA). The performed analytical procedure was described in detail in our previous study (20). Briefly, this software performed 3D endocardial border tracking throughout the cardiac cycle. For sequence analysis, the 16-segment model (apex excluded) was utilized based on the recommendations of the American Society of Echocardiography (19). The time to minimum systolic volume (Tmsv) was measured for each segment and displayed as a curved line throughout the cardiac cycle time. The standard deviation (SD) of the Tmsv was calculated after heart rate variation over 16 segments (Tmsv16-SD%), over 12 segments (Tmsv12-SD%), and over six segments (Tmsv6-SD%). The time difference (Dif) between the shortest and the longest time to the minimum systolic volume was calculated after heart rate variation over 16 segments (Tmsv16-Dif%), over 12 segments (Tmsv12-Dif%), and over six segments (Tmsv6-Dif%).

### Myocardial contrast echocardiography

The standard procedure was described in our previous study (20). Briefly, ultrasound contrast agent Sonovue (Bracco, Milan, Italy) was administered intravenously. Imaging was performed using a low mechanical index under the myocardial perfusion pattern. No side effects were observed in any of the patients. A single perfusion score based on a 16-segment model of LV was assigned to each myocardial segment according to the degree of opacification at the peak contrast effect. Scores were graded as 1 = normal, 2 = reduced, or 3 = absent opacification. The contrast score index (CSI) was calculated by the sum of scores divided by the total number of segments.

### Endpoints

The clinical endpoints comprised 30-day mortality and/or postoperative complications, defined as death within 30 days and/or the occurrence of at least one of the following complications within 7 days after surgery: (a) cardiac (reanimation, cardiogenic shock, pacemaker rhythm/implantation, new supraventricular arrhythmia, new ventricular arrhythmia, hypertensive crisis, intra-aortic balloon pump [IABP], and extracorporeal membrane oxygenation); (b) respiratory (duration of mechanical ventilation ≥24 h, reintubation, tracheotomy, and new pneumonia); (c) neurological (stroke, seizure, sopor, and coma); (d) surgical (reoperation for any reason); (e) infectious (sepsis and deep wound infection); and (f) acute renal failure requiring dialysis (21–23).

### Postoperative follow-up

After the surgical procedure, all the patients were transferred to a cardiac surgical intensive care unit (ICU). The duration in ICU and length of postoperative hospital stays were recorded.

### Statistical analysis

Continuous variables were expressed as means ± SD, or as median and interquartile range (IQR). Categorical variables were expressed as numbers and percentages. For comparing the mean values between the groups, the Student’s *t*-test was used for variables with a normal distribution, and the Mann–Whitney’s *U*-test was used for variables not satisfying the normal distribution. The chi-square test was used to compare categorical variables. The correlation of the Tmsv16-SD% and QRS duration and the relationship among dyssynchrony parameters were assessed using Spearman’ correlation analysis. Univariate logistic regression analysis was used to assess risk factors for post-CABG short-term adverse outcomes. Variables with *P* < 0.10 were included in the multivariate logistic regression model. The odds ratio (OR) from the regression were reported with 95% confidence interval (CI). The area under the receiver-operator characteristic (ROC) curve was calculated. The value closest to the upper left corner of the ROC curve determined the optimal sensitivity and specificity. The best cutoff value for predicting adverse outcome occurrence was determined using the Youden index according to the optimal sensitivity and specificity. The value was carried out to evaluate the prognostic performance of Tmsv16-SD% regarding the short-term clinical outcome. For all tests, *P*-value < 0.05 was significant. All analyses were conducted using SPSS 22.0 software (version 22.0, SPSS Inc., Chicago, IL, USA).

## Results

### Patients’ characteristics

A total of ninety-five 3VD patients were enrolled. Twelve patients (12.6%) showed adverse postoperative short-term outcomes. Some patients had more than one complication. The details about postoperative adverse outcomes were shown in Supplementary Table 1. Baseline characteristics are shown in Table 1. Patients suffering postoperative adverse outcomes had higher level of pre-operative N-terminal pro-brain natriuretic peptide (NT-proBNP) (*P* = 0.021). However, there were no significant differences in the other characteristics.

**TABLE 1:** Baseline characteristics of the study population.

Characteristics	All patients (*n* = 95)	No-adverse outcome group (*n* = 83)	Adverse outcome group (*n* = 12)	*P*-value
Age, years	60.4 ± 8.6	60.8 ± 8.4	57.8 ± 10.3	0.260
Male	80 (84.2%)	71 (85.5%)	9 (75.0%)	0.286
BMI, kg/m^2^	24.7 ± 2.8	24.9 ± 2.9	23.6 ± 1.6	0.160
Hypertension	60 (63.2%)	53 (63.9%)	7 (58.3%)	0.471
Diabetes mellitus	37 (38.9%)	33 (39.8%)	4 (33.3%)	0.464
Unstable angina	30 (31.6%)	26 (31.3%)	4 (33.3%)	0.564
Myocardial infarction	12 (12.6%)	10 (12.0%)	2 (16.7%)	0.468
PCI	17 (17.9%)	15 (18.1%)	2 (16.7%)	0.634
TC, mmol/L	3.9 (3.3, 4.6)	4.0 (3.5, 4.5)	3.6 (2.5, 5.8)	0.377
TG, mmol/L	1.6 (1.0, 1.9)	1.5 (1.0, 1.9)	1.9 (1.2, 2.3)	0.273
HDL, mmol/L	0.9 (0.8, 1.1)	0.9 (0.8, 1.1)	0.9 (0.7, 1.2)	0.561
LDL, mmol/L	2.5 (2.0, 3.0)	2.5 (2.1, 3.0)	2.3 (1.4, 3.8)	0.467
CK-MB, ng/mL	1.5 (1.1, 2.1)	1.5 (1.1, 2.1)	1.4 (0.9, 2.3)	0.942
NT-proBNP, pg/mL	143 (58, 477)	110 (54, 281)	567 (118, 1018)	0.021
QRS duration, ms	88.5 ± 10.9	88.3 ± 11.2	89.2 ± 8.4	0.767
EuroSCORE II, %	1.1 (0.8, 1.7)	1.1 (0.8, 1.6)	1.2 (0.7, 2.5)	0.606

Data are expressed as mean ± standard deviation, median (25 and 75%), and number (percentage). BMI, body mass index; PCI, percutaneous coronary intervention; TC, total cholesterol; TG, total triglyceride; HDL, high density lipoprotein; LDL, low density lipoprotein; WBC, white blood cells; CK-MB, creatine kinase-MB; NT-proBNP, N-terminal pro-B-type natriuretic peptide; EuroSCORE II, European system for cardiac operative risk evaluation II.

### Comparison of echocardiographic characteristics

Two-dimensional echocardiography echocardiographic parameters are summarized in Table 2. There were no significant differences in LVDd, LVDs, LVEDV, LVESV, LVSV, or LVEF between the two groups.

**TABLE 2:** Pre-operative echocardiography parameters.

Parameters	All patients (*n* = 95)	No-adverse outcome group (*n* = 83)	Adverse outcome group (*n* = 12)	*P*-value
**2DE parameters**				
LVDd, mm	49.4 ± 7.1	48.6 ± 6.3	54.5 ± 9.8	0.107
LVDs, mm	32.8 ± 7.7	32.0 ± 7.0	37.8 ± 10.4	0.087
LVEDV, ml	111.6 ± 29.0	110.2 ± 29.4	121.4 ± 25.0	0.213
LVESV, ml	43.5 ± 19.2	42.8 ± 19.1	48.4 ± 19.3	0.305
LVSV, ml	68.0 ± 16.1	67.4 ± 16.5	71.9 ± 13.0	0.372
LVEF, %	62.1 ± 9.6	62.4 ± 9.5	60.3 ± 10.5	0.481
**RT-3DE parameters**				
LVEDV, ml	77.2 ± 20.2	76.1 ± 20.1	83.7 ± 23.8	0.240
LVESV, ml	30.7 ± 12.0	30.0 ± 11.6	35.0 ± 14.1	0.181
LVSV, ml	46.4 ± 12.7	46.0 ± 12.7	48.8 ± 12.3	0.495
LVEF, %	61.1 ± 7.9	61.4 ± 7.9	59.2 ± 8.2	0.372
Tmsv16-SD%, %	2.6 (1.8, 4.4)	2.3 (1.7, 3.8)	5.5 (4.5, 8.1)	<0.001
Tmsv12-SD%, %	2.2 (1.6, 3.6)	2.2 (1.5, 3.1)	4.1 (2.1, 7.7)	0.014
Tmsv6-SD%, %	2.5 (1.6, 3.5)	2.4 (1.5, 3.4)	3.0 (1.9, 6.5)	0.108
Tmsv16-Dif%, %	10.0 (6.7, 17.1)	9.7 (6.4, 13.9)	23.1 (17.4, 24.7)	<0.001
Tmsv12-Dif%, %	7.6 (5.0, 12.2)	7.4 (4.8, 11.4)	13.0 (7.5, 22.8)	0.024
Tmsv6-Dif%, %	6.7 (4.2, 9.7)	6.5 (4.2, 9.3)	8.4 (5.1, 18.3)	0.087
**CSI**	1.13 (1.00, 1.31)	1.13 (1.00, 1.31)	1.34 (1.16, 1.50)	0.010

Data are expressed as mean ± standard deviation and median (25 and 75%). 2DE, two-dimensional echocardiography; LVDd, left ventricular diameter at end-diastole; LVDs, left ventricular diameter at end-systole; LVEDV, left ventricular end-diastolic volume; LVESV, left ventricular end-systolic volume; LVSV, left ventricular stroke volume; LVEF, left ventricular ejection fraction; RT-3DE, real-time three-dimensional echocardiography; Tmsv16-SD%, standard deviation of time to minimum systolic volume calculated after heart rate variation over 16 segments; Tmsv12-SD%, over 12 segments; Tmsv6-SD%, over 6 segments; Tmsv16-Dif%, time difference between the shortest and the longest time to minimum systolic volume calculated after heart rate variation over 16 segments; Tmsv12-Dif%, over 12 segments; Tmsv6-Dif%, over 6 segments; CSI, contrast score index.

**FIGURE 1:**
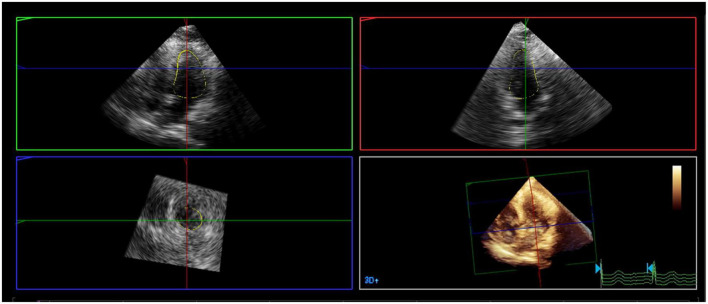
A mathematical full-volume model of the left ventricle by real-time three-dimensional echocardiography.

**FIGURE 2:**
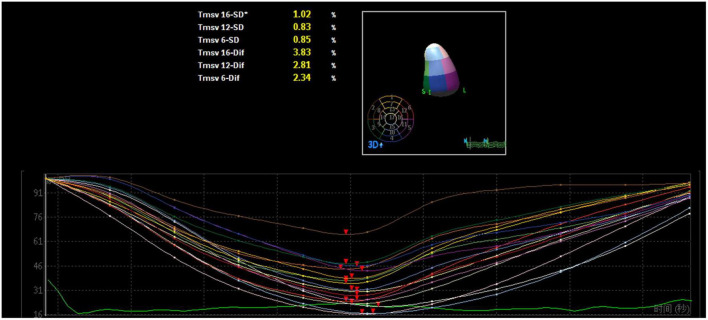
Three-dimensional echocardiography assessment of left ventricular dyssynchrony of a patient with normal intraventricular synchrony. The time–volume curves and the time of reaching the trough are consistent and overlapping.

**FIGURE 3:**
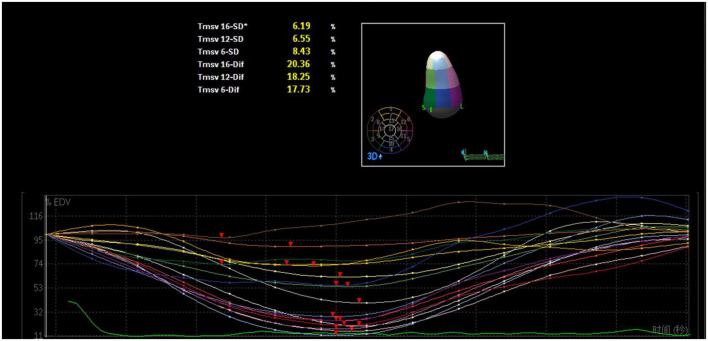
Three-dimensional echocardiography assessment of left ventricular dyssynchrony of a patient with intraventricular dyssynchrony. The time-volume curves are not consistent and lagged, and the disordered time-volume curves signify LV dyssynchrony.

A mathematical full-volume model of the LV was obtained through RT-3DE (Figure 1). Patients with good postoperative outcomes mostly showed consistent and overlapping curves (Figure 2). However, patients with adverse postoperative outcomes mostly presented with lagged time-volume curves (Figure 3). Patients with adverse postoperative outcomes had significantly higher LV dyssynchrony parameters obtained through RT-3DE, including Tmsv16-SD% (*P* < 0.001), Tmsv12-SD% (*P* = 0.014), Tmsv16-Dif% (*P* < 0.001), and Tmsv12-Dif% (*P* = 0.024).

The myocardial perfusion index, CSI, was different between the two groups (*P* = 0.010).

The correlations among the dyssynchrony parameters were significant (*P* < 0.001). However, there was no correlation between Tmsv16-SD% and QRS duration (*r* = 0.073, *P* = 0.500) (Supplementary Figure 1).

### Predictors for postoperative occurrence of short-term adverse outcomes

The univariate logistic regression analysis identified the following parameters with *P*-value < 0.10: NT-proBNP (*P* = 0.045), LVDd (*P* = 0.012), Tmsv16-SD% (*P* = 0.002), Tmsv12-SD% (*P* = 0.015), Tmsv6-SD% (*P* = 0.028), Tmsv16-Dif% (*P* = 0.015), Tmsv12-Dif% (*P* = 0.060), Tmsv6-Dif% (*P* = 0.017), and CSI (*P* = 0.014) (Table 3). Due to a strong internal correlation between the LV synchronization indexes, we only included Tmsv16-SD%, the most representative synchronization index, (24) and the other variables in the multivariate model. The multivariate logistic regression analysis indicated that Tmsv16-SD% (OR 1.362, 95% CI 1.090–1.702, *P* = 0.006) was independently associated with the occurrence of postoperative short-term adverse outcomes (Table 4).

**TABLE 3:** Univariate logistic regression analyses of variables predictive of short-term adverse outcome occurrence after CABG.

Variables	Odds ratio	95% CI	*P*-value
**Baseline characteristics**			
Age, years	0.960	0.894–1.031	0.260
Male	1.972	0.466–8.347	0.356
BMI, kg/m^2^	0.856	0.689–1.064	0.162
Hypertension	0.792	0.231–2.716	0.711
Diabetes mellitus	0.758	0.211–2.720	0.670
Unstable angina	1.096	0.303–3.969	0.889
Myocardial infarction	1.460	0.279–7.646	0.654
PCI	0.907	0.180–4.572	0.906
TC, mmol/L	1.053	0.660–1.680	0.829
TG, mmol/L	1.035	0.576–1.859	0.908
HDL, mmol/L	0.599	0.042–8.467	0.704
LDL, mmol/L	1.046	0.549–1.991	0.891
CK-MB, ng/mL	0.977	0.467–2.044	0.952
NT-proBNP, pg/mL	1.001	1.000–1.002	0.045
QRS duration, ms	1.007	0.952–1.065	0.807
**2DE parameters**			
LVDd, mm	1.108	1.023–1.201	0.012
LVDs, mm	1.089	1.012–1.172	0.122
LVEDV, ml	1.013	0.993–1.034	0.214
LVESV, ml	1.014	0.985–1.045	0.342
LVSV, ml	1.017	0.980–1.056	0.370
LVEF, %	0.977	0.917–1.041	0.478
**RT-3DE parameters**			
LVEDV, ml	1.017	0.989–1.045	0.241
LVESV, ml	1.032	0.985–1.082	0.185
LVSV, ml	1.016	0.971–1.064	0.491
LVEF, %	0.966	0.895–1.042	0.369
Tmsv16-SD%, %	1.405	1.129–1.749	0.002
Tmsv12-SD%, %	1.300	1.052–1.608	0.015
Tmsv6-SD%, %	1.392	1.036–1.871	0.028
Tmsv16-Dif%, %	1.063	1.012–1.116	0.015
Tmsv12-Dif%, %	1.058	0.998–1.122	0.060
Tmsv6-Dif%, %	1.153	1.026–1.295	0.017
**CSI**	15.248	1.735–133.990	0.014

CABG, coronary artery bypass grafting; BMI, body mass index; PCI, percutaneous coronary intervention; TC, total cholesterol; TG, total triglyceride; HDL, high density lipoprotein; LDL, low density lipoprotein; CK-MB, creatine kinase-MB; NT-proBNP, N-terminal pro-B-type natriuretic peptide; 2DE, two-dimensional echocardiography; LVDd, left ventricular diameter at end-diastole; LVDs, left ventricular diameter at end-systole; LVEDV, left ventricular end-diastolic volume; LVESV, left ventricular end-systolic volume; LVSV, left ventricular stroke volume; LVEF, left ventricular ejection fraction; RT-3DE, real-time three-dimensional echocardiography; Tmsv16-SD%, standard deviation of time to minimum systolic volume calculated after heart rate variation over 16 segments; Tmsv12-SD%, over 12 segments; Tmsv6-SD%, over 6 segments; Tmsv16-Dif%, time difference between the shortest and the longest time to minimum systolic volume calculated after heart rate variation over 16 segments; Tmsv12-Dif%, over 12 segments; Tmsv6-Dif%, over 6 segments; CSI, contrast score index; CI, confidence interval.

**TABLE 4:** Multivariate logistic regression analyses of variables predictive of short-term adverse outcome occurrence after CABG.

Variables	Odds ratio	95% CI	*P*-value
LVDd, mm	1.082	0.993–1.179	0.072
NT-proBNP, pg/mL	1.000	0.998–1.002	0.415
Tmsv16-SD%, %	1.362	1.090–1.702	0.006
CSI	3.831	0.379–38.771	0.228

CABG, coronary artery bypass grafting; LVDd, left ventricular diameter at end-diastolic; NT-proBNP, N-terminal pro-B-type natriuretic peptide; Tmsv16-SD%, standard deviation of time to minimum systolic volume calculated after heart rate variation over 16 segments; CSI, contrast score index; CI, confidence interval.

**FIGURE 4:**
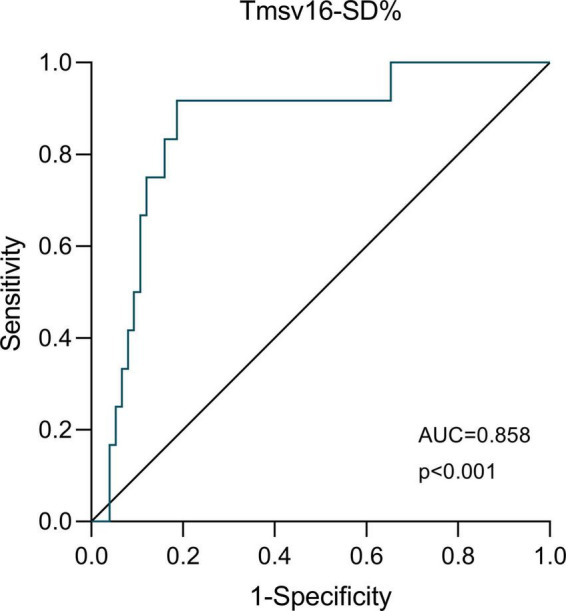
The receiver-operator characteristic (ROC) analysis. ROC analysis for the ability of Tmsv16-SD% to discriminate patients with or without the occurrence of short-term adverse outcomes after coronary artery bypass grafting.

The ROC curve analysis of Tmsv16-SD% as a predictor of adverse short-term outcome occurrence is shown in Figure 4. We demonstrated that the optimal Tmsv16-SD% cutoff value for short-term adverse clinical outcomes was 4.1%, with an area under the curve (AUC) of 0.858, the sensitivity of 91.7%, and the specificity of 81.3%.

### Comparison of postoperative follow-up characteristics according to Tmsv16-SD%

As shown in Table 5, 3VD patients with preserved or mildly reduced LVEF were grouped according to the Tmsv16-SD% cutoff value. Patients with Tmsv16-SD%>4.1% (*n* = 24) before CABG had a longer postoperative stay in ICU (*P* < 0.001) and hospital (*P* = 0.043).

**TABLE 5:** Comparison of postoperative characteristics according to Tmsv16-SD%.

Characteristics	All (*n* = 95)	Tmsv16-SD% ≤4.1% (*n* = 71)	Tmsv16-SD% >4.1% (*n* = 24)	*P*-value
ICU stay, day	2 (1, 3)	2 (1, 2)	3 (2, 5)	<0.001
Post-operative, day	16 (13, 20)	15 (13, 19)	17 (14, 22)	0.043

Data are expressed as mean ± standard deviation and median (25 and 75%). ICU, intensive care unit; Tmsv16-SD%, standard deviation of time to minimum systolic volume calculated after heart rate variation over 16 segments.

## Discussion

This study revealed that 3VD patients with preserved or mildly reduced LVEF undergoing CABG who experienced postoperative short-term adverse outcomes had a higher preoperative LV systolic dyssynchrony. The parameter Tmsv16-SD% obtained from RT-3DE was identified as an independent risk factor. Thus, RT-3DE was a useful additional method for the risk classification in 3VD patients with preserved or mildly reduced LVEF before CABG. To our knowledge, this is the first study which focus on predicting adverse postoperative short-term outcomes occurrence of 3VD patients with preserved or mildly reduced LVEF, based on RT-3DE measurement.

It is crucial to identify individuals at high risk before undergoing CABG surgery. Accordingly, the modified EuroSCORE II has been developed for risk assessment and treatment guidance. However, it still shows defective discrimination ability in terms of sensitivity and accuracy (25). A similar unsatisfactory result was also found in our study, given that the EuroSCORE II was similar between the two groups.

Patients with preserved or mildly reduced LVEF were included in our study, and the measured LVEF in both groups with different outcomes was comparable. This volumetric approach mainly reflecting the change in LV morphology was not able to adequately describe the early change of myocardial dysfunction. It is important to identify additional indicators that can supplement risk stratification. The impairment of LV systolic synchrony is prevalent among CAD patients, and it can also occur in those patients with preserved or mildly reduced LVEF (26–28). In our study, the LV dyssynchrony parameters, including Tmsv16-SD%, Tmsv12-SD%, Tmsv16-Dif%, and Tmsv12-Dif% were higher in the group with short-term adverse outcomes. These LV dyssynchrony parameters might be more sensitive indicators than LVEF in an early stage of LV dysfunction in 3VD patients. Furthermore, LV systolic dyssynchrony is kind of LV mechanical dysfunction (LVMD), which indicates a difference in the timing of mechanical contraction or relaxation between different segments and might precede any changes in overt regional wall motion or global systolic abnormalities (28). There was also no correlation between Tmsv16-SD% and QRS duration in this study, which suggested that the increased dyssynchrony resulted from mechanical disturbance might be more sensitive than QRS duration. The similar findings were reported by Wang H et al. (29). When coronary artery stenosis or blocking occurs progressively, based on reduced myocardial oxygen and blood flow, myocardial hypoperfusion and ischemia occur (16). Cardiomyocytes and myocardial contractile proteins are damaged and myocardial metabolism is disturbed. Thus, myocardial deformation property as reflected by LV synchrony is decreased (30). Another pathological process, the decrease in coronary flow velocity due to the obstructed segments of the coronary artery, may also lead to synchronous changes due to impaired microcirculation. Myocardial microvascular ischemia can result from local inflammation and vascular rarefaction demonstrated in CAD (31). In our study, an accordant result regarding the myocardial perfusion change was found. The CSI was higher, indicating more severe hypoperfusion in the adverse outcome group. Some studies have also advocated that LV mechanical activation is triggered by the rapid propagation of electrical signals *via* the specialized conduction system, while the early impaired myocardial perfusion in CAD patients may damage the synchronization of LV mechanical activity by reducing electromechanical coupling and LV coordination, resulting in a change of LV myocardial mechanics (30, 32).

Moreover, preoperative LV systolic dyssynchrony appeared to play an important role in the occurrence of post-CABG short-term adverse outcomes. The most representative parameter, Tmsv16-SD%, was significantly associated with the occurrence of short-term adverse outcomes, according to the multivariate regression analysis. This indicated the significance of LVMD in CAD patients with preserved or mildly reduced LVEF. Fudim et al. (33) also found a close link between LV dyssynchrony and adverse clinical outcome by single-photon emission computed tomography. To our knowledge, data on predicting adverse postoperative short-term outcomes occurrence of CAD patients with preserved or mildly reduced LVEF, based on RT-3DE measurement, are still scarce.

The LV dyssynchrony parameters for different segments in this study were higher than those in a community-based population, (24) but lower than those in patients with severe LV dysfunction (34). In addition, our findings indicated that Tmsv16-SD% above 4.1% had a sensitivity of 91.7% and a specificity of 81.3% for predicting post-CABG short-term adverse outcome occurrence. When patients were dichotomized according to Tmsv16-SD%, those patients with inferior synchrony were more likely to spend more time in ICU and hospital after operation. Tmsv16-SD% might serve as a sensitive indicator and may provide an additional diagnostic standard in patients with preserved or mildly reduced LVEF before CABG operation. This indicator will alert clinicians to which patients need more attention and tend to have more postoperative complications. These procedures allow some patients to avoid short-term adverse postoperative outcomes and suffer less physical damage or economic loss.

Echocardiography is one of the cornerstones of cardiac function assessment. Previous studies have reported that conventional echocardiography has some inherent limitations, including the inability to image the whole structure simultaneously, geometric assumptions, and the possibility of imperfect alignment (35, 36). For RT-3DE, the endocardial surface can be automatically detected throughout the cardiac cycle, allowing accurate identification of the timing of the end of ejection; thus, global LV systolic function and mechanical dyssynchrony can be evaluated simultaneously. It is possible to quantify global and regional cardiac deformation non-invasively. An addition of echocardiographic assessment of cardiac function would enhance the predictive accuracy and thus would facilitate clinical decision-making.

Our study had several limitations. First, the limited size of the present study population may have affected the power of the present analysis. The presented study was designed as a single-center study; thus, validation from multiple centers could not be obtained. Therefore, to support the viewpoint from this study, larger studies are needed with a higher number of patients and with multiple centers. Second, although patients with poor image quality were excluded as much as possible, RT-3DE imaging could also have led to some errors in the volume measurement due to its definition of the endocardial border.

## Conclusion

The current study demonstrated an independent association between pre-operative Tmsv16-SD% and the occurrence of short-term adverse outcomes after CABG in 3VD patients with preserved or mildly reduced LVEF. Tmsv16-SD% obtained by RT-3DE may be useful for additional evaluation of LV systolic function and can improve echocardiographic evaluation levels in pre-operative risk classification.

## Data availability statement

The raw data supporting the conclusions of this article will be made available by the authors, without undue reservation.

## Ethics statement

The studies involving human participants were reviewed and approved by the Institutional Review Board of The First Affiliated Hospital of Sun Yat-sen University. The patients/participants provided their written informed consent to participate in this study.

## Author contributions

F-JY, WL, and JL contributed towards the conception and design of the study. JL collected data of clinical trials and drafted the manuscript together with RF and C-LL. Y-QL and D-HL revised and edited the final version of the manuscript for important intellectual content and gave the technical support. JL and RF contributed equally to the acquisition of data, e.g., RF contributed in examination, and JL contributed towards the to patient collection and gave technical support. JL and RF performed data analysis together, including imaging, computation and statistical analysis, and edited the manuscript together. All authors read and approved the final manuscript.
