# Supplementary material for: Predictive value of left ventricular dyssynchrony for short-term outcomes in three-vessel disease patients undergoing coronary artery bypass grafting with preserved or mildly reduced left ventricular ejection fraction
Source: Front Cardiovasc Med. 2022 Nov 17;9:1036780. doi: 10.3389/fcvm.2022.1036780 (PMC9714482; doi:10.3389/fcvm.2022.1036780)
Supplement: Supplementary file 1 [file Data_Sheet_1.docx]

Supplementary Material

**Predictive Value of Left Ventricular Dyssynchrony for Short-term Outcomes in Three-vessel Disease Patients Undergoing Coronary Artery Bypass Grafting with Preserved Left Ventricular Ejection Fraction**

**Supplementary** **Table 1.** The details of the postoperative adverse outcomes for each patient

| patient | mortality and/or postoperative complications |
| --- | --- |
| 1 | mechanical ventilation for ≥ 24 h, new pneumonia |
| 2 | dead (mainly due to cardiogenic shock) |
| 3 | newly developed supraventricular arrhythmia (atrial fibrillation) |
| 4 | newly developed supraventricular arrhythmia (atrial fibrillation), newly developed ventricular arrhythmia (ventricular premature beat) |
| 5 | temporary pacemaker, deep wound infection |
| 6 | intra-aortic balloon pump |
| 7 | newly developed supraventricular arrhythmia (supraventricular tachycardia), intra-aortic balloon pump, mechanical ventilation for ≥ 24 h, acute renal failure requiring continuous renal replacement therapy |
| 8 | intra-aortic balloon pump |
| 9 | intra-aortic balloon pump |
| 10 | intra-aortic balloon pump, pericardial tamponade, required reintubation, tracheotomy, acute renal failure requiring continuous renal replacement therapy |
| 11 | newly developed supraventricular arrhythmia (atrial fibrillation) |
| 12 | newly developed supraventricular arrhythmia (atrial fibrillation), newly developed ventricular arrhythmia (ventricular premature beat) |

**Supplementary Figure 1.**


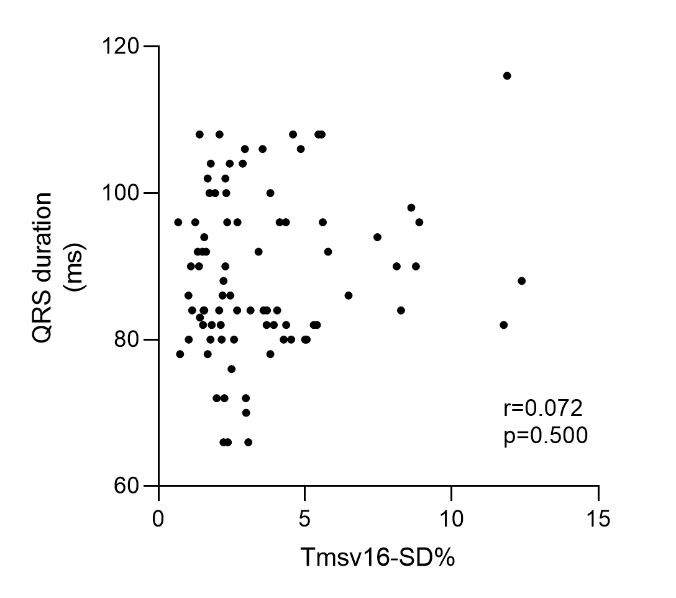


**Figure Legends**

Supplementary Figure 1. There was no correlation between Tmsv16-SD% and QRS duration (r = 0.073, P = 0.500).
